# *Aurelia aurita* as a Model for Ecotoxicologically Assessing Food Additives: 2-Methyl-1-phenylpropan-2-ol and 1-Phenylethan-1-ol

**DOI:** 10.3390/toxics13070572

**Published:** 2025-07-07

**Authors:** Borja Mercado, Borja Vila, Luis Roca-Pérez, Neus Duran-Giner, Rafael Boluda-Hernández, Oscar Andreu-Sánchez

**Affiliations:** 1 Department of Biomedical Sciences, Faculty of Health Sciences, European University of Valencia, Paseo de La Alameda, 7, 46010 Valencia, Spain; borja.mercado@universidadeuropea.es (B.M.); evadelesneus.duran@universidadeuropea.es (N.D.-G.); 2 Departamento de Biología Vegetal, Facultad de Farmacia, Universitat de València, 46100 Burjassot, Spain; borjavilapiqueras@gmail.com (B.V.); rafael.boluda@uv.es (R.B.-H.); oscar.andreu@uv.es (O.A.-S.)

**Keywords:** cnidarian, ecotoxicology, additives, trophic levels, bioassays

## Abstract

Industry currently generates numerous substances, such as food additives, whose environmental impacts, particularly in marine environments, remain inadequately assessed. This study employed *Aurelia aurita* for the first time as a model organism to evaluate the toxicity of such compounds. The main goal was to evaluate the toxicity of two food additives, 2-methyl-1-phenylpropan-2-ol (S1) and 1-phenylethan-1-ol (S2), on *A. aurita* ephyrae, comparing the results with other organisms representing different trophic levels, specifically the alga *Phaeodactylum tricornutum* and the crustacean *Artemia salina*. Acute toxicity tests were conducted on each organism. In *A. aurita*, S1 exhibited high toxicity (LC_50_ ≈ 10 mg/L), while S2 had lower toxicity (LC_50_ ≈ 80 mg/L). The pulsation frequency data for *A. aurita* revealed that S1 initially increased the pulsation rates at lower concentrations (maximum at 10 mg/L), followed by a significant decrease at higher concentrations. Conversely, S2 showed a steady decrease in pulsation rates up to 10 mg/L, with a slight increase at concentrations of 15, 20, and 25 mg/L. The results demonstrate varying sensitivities to the toxic effects of the two compounds across different trophic levels, with *A. aurita* ephyrae being the most sensitive. This suggests the potential efficacy of jellyfish as novel ecotoxicological models due to their heightened sensitivity, enabling the detection of lower contaminant concentrations in test samples. Further research is required to enhance the efficiency of ecotoxicological assays using this model.

## 1. Introduction

The 21st century has been marked by exponential growth in industrial and technological development, leading to significant increases in the production and use of chemical compounds. These, coupled with the unsustainable rise in the global human population, have resulted in a concerning consequence: the excessive increase in anthropogenic pollutants in the environment [1]. Among these pollutants, chemical additives stand out, which are widely used across various industrial and domestic sectors [2].

Additives are compounds designed to control potentially harmful organisms such as bacteria, fungi, algae, and insects among other applications. They are present in everyday products, such as pesticides, herbicides, disinfectants, and preservatives, and are used in various fields including agriculture, human medicine, veterinary medicine, the food industry, and environmental management. They are also used to improve the physical, chemical, or biological properties of different products. They are found as we mentioned earlier in food, cosmetics, plastics, and many other daily use items [3].

Modern additives are designed to target specific organisms; however, they often are toxic to many other living organisms that are not the intended target. Moreover, the target organisms often coexist in their natural habitat with other, more sensitive species [4]. Chemical additives can have carcinogenic, toxic, or endocrine-disrupting properties. Additionally, some of them are not biodegradable, posing a danger to the environment in cases of emissions, spills, and leaks [5].

Among these additives, 2-methyl-1-phenylpropan-2-ol (S1) and 1-phenylethan-1-ol (S2) are common in industrial processes. The first one was selected in this study due to its unique steric and electronic properties, which made it an ideal model in the context of reactivity and chemical analysis. These characteristics make it a suitable and versatile reagent or intermediate in a wide range of organic transformations [6] including oxidation to form ketones or carboxylic acids and esterification to produce esters, mainly used in perfumery. The second one is a chiral benzylic alcohol widely used as a fragrance and flavoring agent. It is also an important industrial intermediate in styrene production and the main phase I metabolite of ethylbenzene. Despite its relevance, the available toxicological data are limited and largely based on in vitro studies. Its structural features and biological significance make it a suitable model compound for further investigation [7]. Both 1-phenylethanol and S1 are widely used additives in such applications. Their frequent use, combined with their structural features (aromatic alcohols with differing steric profiles), made them ideal candidates for assessing potential reactivity and behavior under the studied conditions. This selection allowed us to better understand the implications of their use in real-world formulations and their relevance in both human and environmental exposure [8].

Marine ecosystems are probably the most affected by the current concerning situation due to the incessant increase in anthropogenic pressure, resulting from the increased release of new pollutants through various activities, many of which are directly discharged into seas and oceans, in addition to indirect emissions such as pollutant discharges into non-endorheic river basins. To date, no environmental monitoring data have been found regarding the presence or concentration levels of S1, and only Dao et al. (2014) [9] reported some environmental concentrations for S2. A comprehensive review of the available scientific literature reveals that these compounds have primarily been studied in the context of chemical synthesis and laboratory-scale reactions. This gap highlights the need for further investigation to assess their potential environmental distribution and impact. In this context, where these compounds pose potential threats to the biodiversity and functionality of these systems both locally and globally, the lack of adequate infrastructure for wastewater treatment and the irresponsible disposal of industrial and urban waste are key factors in the accumulation of these pollutants in water [10,11]. When these substances enter water, due to their high resistance to biodegradation, they disperse through seas and oceans, not limited to direct discharge areas, and are transported over long distances by ocean currents [12]. The accumulation of chemicals and biocides in marine sediments and water columns has emerged as a critical problem, with potentially severe impacts endangering the health of multiple organisms and disrupting marine ecosystems [13]. Moreover, there is growing concern about the secondary effects of these chemicals on human health, especially through bioaccumulation and biomagnification in the marine food chain [3].

Due to the presence and potential impact of marine pollutants on both humans and wildlife, the importance of monitoring and testing the fate and effects of these chemicals has long been recognized [14,15]. This is one of the main goals of ecotoxicology assays. However, in ecotoxicology there is an urgent need to identify new model organisms for their use in the development of sensitive and reliable methods for laboratory testing. Gelatinous zooplankton are not typically included in routine ecotoxicological studies, despite the growing recognition over the past few decades of their crucial role in maintaining the balance of marine ecosystems.

Jellyfish, which comprise part of the gelatinous zooplankton, play a critical role in marine ecosystems by preying on planktonic organisms such as crustaceans, copepods, and fish larvae. They, in turn, are consumed by species like sea turtles, sunfish, and seabirds. As predators, jellyfish can significantly alter plankton composition and affect fishery yields through dietary overlap and direct predation. Jellyfish blooms are episodic events driven by complex physical, behavioral, and physiological processes, often exacerbated by human activities like overfishing, eutrophication, and changes in estuarine circulation. These factors have prompted the exploration of using the ephyra stage of the Scyphozoan jellyfish *Aurelia aurita* (common moon jellyfish) as an innovative model organism in marine ecotoxicology [16,17].

Given the reasons mentioned above, the ephyrae of *A. aurita* were selected to test compounds S1 and S2, which are commonly used as additives in the food industry as well as in the perfume industry. To compare the effects and sensitivities of these food additives on scyphomedusae, the compounds were also tested on two additional marine organisms, *P. tricornutum* (diatom) and *A. salina* (crustacean). The primary objective of these assays was to evaluate the toxicity of the tested compounds in marine invertebrates and their potential impact on the marine food chain.

## 2. Materials and Methods

Acute toxicity was assessed by exposing organisms to different concentrations of the test substances for 24, 48, or 72 h [18] with three replicates for each test. The LC_50_ values were calculated based on the survival, mortality, and pulsation frequency in *A. aurita*, following the protocols of Faimali et al. (2014) and Mercado Casares et al. (2023) [16,19]. Pulsation frequency was suggested by Faimali et al. (2014) as an easily measurable endpoint and can be coupled with an acute one (immobilization) [14].

### 2.1. Tested Additives

Both additives were provided by Enamine Ltd. Company, Riga, Latvia, with a certified purity of 95%. To ensure the stability of the compounds across time and concentrations, the higher-concentration prepared dilutions in each bioassay were analyzed using a high-performance liquid chromatography system (Agilent™ 1220–1260 Infinity^®^ Detector Diode Array, Santa Clara, California, USA) according the procedures described in [20,21]. According to ECHA registration dossiers, both compounds have a water solubility higher than the concentration used in our study [22,23]. The main chemical characteristics are presented in Table 1. No carrier or solvents were used in the assays. The compound S1 has demonstrated functionalities as a food additive, a cleaning product, and in perfumery cosmetics. Currently, despite being considered a toxic compound in cases of inhalation and ingestion in humans (H322 and H302), it is in the process of registration under the ecotoxicity section of the REACH regulation due to the insufficient and inconclusive information for classification and, therefore, authorization. Currently, there is no consensus on the classification of compound S1. According to Article 10 of REACH, ECHA records indicate that it is synthesized or imported into the EU in quantities between 10 and 100 t per year, and its use in food, medicines, pesticides, or biocidal products is not recommended [23]

The compound S2 (1-phenylethan-1-ol) was designed as an additive for perfumes, cleaning products, and pharmaceuticals.

The toxicity ranges selected for classifying the degree of toxicity for the three models were chosen based on the Regulation (*EC*) No. 1272/2008 [24] on the classification, labelling, and packaging of substances and mixtures (*CLP* Regulation). The EC_50_ values were ranked into four toxicity categories (cat): cat 1, highly toxic substances with E(L)C_50_ ≤ 1 mg/L; cat 2, from >1 to ≤10 mg/L (medium toxicity); cat 3, from >10 to ≤100 mg/L (low toxicity); and cat 4 for non-toxic substances with E(L)C_50_ > 100 mg/L. This classification has already been used by other authors in similar studies [25,26].

### 2.2. Artemia Salina Acute Test

The assay was based on Artoxkit M™ (Microbiotests Inc., Gent, Belgium) and conducted according to its standard operational procedure. The company generates the cyst from their own live stock. All tests used cysts from a single batch. Artificial seawater was prepared using the preconcentrated media supplied with the kit. This water was used for the incubation of the cysts, the dilution of the compounds, and the execution of the toxicity assays. All tests were conducted with *Artemia* nauplii at the II–III-instar stage, aged between 24 and 48 h post-hatching. A small quantity of cysts was placed in a beaker with standard saline water at 25 °C and incubated in a climatic chamber at 25 °C (±2 °C) for 30 h, ensuring continuous aeration via an air stone and constant illumination provided by a 4000 lux growth lamp.

Serial dilutions of the test compounds were prepared in the artificial seawater supplied with Artoxkit M™ in concentration series of 1000, 700, 500, 300, and 100 mg/L. Depending on the initial test results, further dilutions were prepared to achieve mortality results close to the LC_50_, thus aiming for more precise results. Once the different concentrations of the compounds were prepared, they were placed in 24-well microplates (Nunc^®^) with a volume of 3 mL per well. The plates were covered to prevent evaporation and sealed with Parafilm™ to ensure airtightness. One 24-well plate was used for each compound, and a second 24-well plate was used as a blank control.

Each well was filled with 1 mL of dilution, using the first two wells as washing wells to avoid over-diluting the sample and thus minimize errors when transferring the *Artemia* nauplii to the plates. Twenty nauplii were added to each concentration using a transfer pipette and incubated at 25 °C (±2 °C) in darkness for 24 h. After this period, an initial mortality count was conducted using a binocular magnifying glass, followed by a subsequent incubation period until 48 h, at which point a second mortality count was performed. In all cases, control mortality did not exceed 10%. In accordance with the test guidelines provided by the supplier, nauplii were considered dead when immobility was observed for a continuous 10 s interval. Since this criterion was used as a surrogate for mortality, LC_50_ values were determined based on this definition.

### 2.3. Phaeodactylum Tricornutum Acute Test

The assay was conducted following the Marine Algaltoxkit™ protocol by Microbiotests Inc. (Gent, Belgium), designed according to the ISO 10253:2016 guideline [27] International Organization for Standardization: Geneva, Switzerland, 2016. The kit included all necessary materials for the assays, including vials with the inoculum of *P. tricornutum* (generated from an axenic stock culture), which were stored at +5 °C (±2 °C) in darkness until use. The algal growth medium was prepared from the pre-concentrates provided in the kit (final concentrations in the medium: NaCl, 6.4 g/L; KCl, 840 mg/L; CaCl_2_·2H_2_O, 1670 mg/L; MgCl_2_·6H_2_O, 4600 mg/L; MgSO_4_·7H_2_O, 5580 mg/L; NaHCO_3_, 170 mg/L; H_3_BO_3_, 30 mg/L). This medium was enriched with a nutrient solution provided in the kit to facilitate algal growth. The medium was used for incubation, compound dilution, and toxicity testing. Prior to the assay, a pre-incubation of the *P. tricornutum* inoculum was conducted. A 25 mL vial was emptied into a 50 mL beaker, and the vial was rinsed twice with 7.5 mL of the medium to retrieve as much inoculum as possible. The beaker was sealed with Parafilm^®^, and the final 40 mL dilution was incubated for 72 h at 20 °C (±2 °C) under 4000 lux illumination as specified by the ISO standard. After the incubation period, measurements were taken with a benchtop VIS spectrophotometer (Jenway™ model 6305, Staffordshire, UK) at 670 nm using 100 mm glass cuvettes, ensuring a 10 s agitation prior to measurement, confirming a cell concentration of 1 × 10^6^ cells/mL. With the culture at the required density, 75 mL Erlenmeyer flasks were used, each containing 50 mL of the toxicant dilution and algal inoculum to achieve an initial density of 1 × 10^4^ cells/mL.

Serial dilutions of the toxicants were prepared in the artificial seawater for algal growth, with concentration series of 500, 250, 125, 62.5, and 31.25 mg/L Based on the initial test results, dilutions were adjusted to achieve mortality results close to the LC_50_, aiming for more precise outcomes.

### 2.4. Aurelia Aurita Acute Test

**Figure 1:**
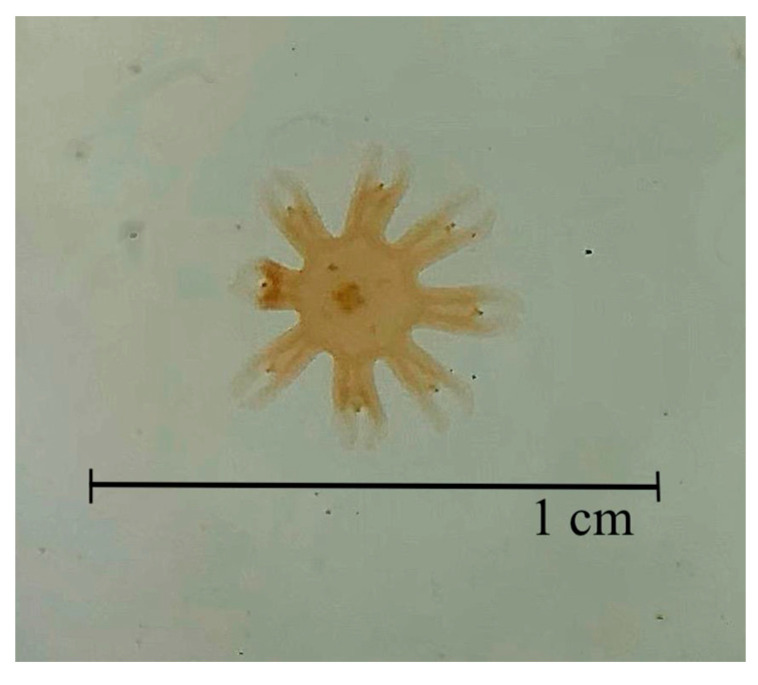
*Aurelia aurita* ephyrae after the strobilation.

This assay was adapted from the guidelines for the protocol by Faimali et al. (2014) and Mercado et al. (2023) [16,19]. The ephyrae of *A. aurita* were supplied by the Oceanogràfic of Valencia (Valencia, Spain). They were used 24 h post-strobilation; thus, no prior preservation was necessary. All tests utilized ephyrae generated from the same polyp population and within the same size range (Figure 1).

Dilutions were prepared based on the LC_50_ values obtained from the *A. salina* assays. However, due to the high sensitivity of *A. aurita* to the compounds, lower concentrations (below 50 mg/L) were tested: 50, 25, 15, 10, and 2 mg/L. The dilutions were tested using 20 mL capacity cell culture multiwell plates with lids to prevent evaporation. The plates were sealed with Parafilm^®^ (Amcor™, Lancaster, WI, USA) to ensure airtightness and prevent salinity variations. Each well was filled with 10 mL of the compound dilution, and two ephyrae were transferred to each well. The wells were sealed with Parafilm^®^ and maintained in a thermostatic refrigerator at 20 °C (±2 °C) under dark conditions.

After 24 and 48 h, two parameters were assessed: pulsation frequency and mortality rate. Pulsation frequency was defined as the number of pulsations performed by an ephyra within a 60 s interval and is expressed as the percentage change relative to the control group. All observations were made by means of a ×20 stereomicroscope model Stemi 305 trino equipped with ring LED illuminator (Zeiss™, Jena, Deutchland, Germany). The mortality rate was calculated as the percentage of dead ephyrae at each concentration relative to the control. A video (Video S1) demonstrating the pulsation behavior of *Aurelia aurita* ephyrae following strobilation is available in the Supplemental Materials.

### 2.5. Statistical Analysis

Finally, the LC_50_ values were estimated using Probit regression analysis, a method commonly applied in ecotoxicological studies for its robustness and comparability across similar datasets [19,27]. The data met the assumptions for Probit analysis, and the model provided a satisfactory fit to the observed responses [28]. Also, LC_10_ values were calculated; however, these values are not recognized for classification and labeling purposes under the GHS, REACH, or CLP regulations; they are presented in this study for scientific interpretation. These values are included to enhance the toxicological profile discussed in this manuscript, but they are not intended for regulatory use or product labeling. In addition, to compare the differences between groups, ANOVA tests were conducted. Statistical analyses were performed using RStudio 12.0. A one-way ANOVA was conducted to evaluate differences among treatments, with statistical significance set at α = 0.05.

### 2.6. Environmental Risk Assessment (ERA)

The environmental risk characterization for chemicals in the EU relies on the ratio of the predicted environmental concentration (PEC) to the predicted no-effect concentration (PNEC). This approach is detailed in the guidance on information requirements and chemical safety assessment (ECHA, 2008) [29] and formalized within the European Union System for the Evaluation of Substances (EUSES) (EU, 2008) [30,31]. Considering the environmental concentrations, we assessed the risk following the mentioned approach. The PNECs were calculated from the LC_10_ values using an assessment factor (AF) of 1000. The LC_10_ can be considered a proxy for the no-observed-effect concentration (NOEC) in acute tests, providing insight into sublethal toxicity. This analysis could be considered as a first approach for an environmental risk assessment [32].

## 3. Results

The toxicity assay results revealed distinct sensitivity levels among the three tested organisms. The LC_50_ values were determined for each species, providing insight into their respective tolerances to the compounds studied, being 223 mg/L for S1 and 384.4 mg/L for S2 (Table 2). The LC_50_ for *A. salina* was calculated as 577.9 mg/L for S1 and 623.30 mg/L for S2, while *P. tricornutum* exhibited an LC_50_ of 89.0 mg/L and 452.9 mg/L for S1 and S2, respectively. Notably, A. aurita showed the highest sensitivity, with an LC_50_ of 2.5 mg/L for S1 and 77 mg/L for S2. A concentration–response plot for each of the three assays is available in the Supplemental Materials (Figure S1). These findings highlight the different sensitivities of the model marine organisms considered after acute exposure to S1 and S2 food additives. The results are based on measured concentrations. However, if evidence is available to demonstrate that the concentration of the test substance has been satisfactorily maintained within ±20 percent of the nominal initial concentration throughout the test, then the results can be considered to be based on the nominal initial values [33]. The different concentrations of each additive tested with HPLC remained almost constant over time, with a variation in the bioassay concentration at the end of the assay lower than 5%, ensuring that the amount of the studied compounds was stable throughout the assay. This consistency confirms the reliability of the measured concentrations throughout this study (results on the concentration variation are available in Supplementary Table S1).

The results of the analysis of variance (ANOVA) in Table 3 indicate that there are significant differences in the LC_50_ among the three tested organisms for the S1 and S2 compounds (*p* < 2 × 10^−16^). The sum of squares (Sum Sq) and the mean squares (Mean Sq) for the organism factor were high for both additives, while the mean square was close to 0. These results suggest that the observed differences in the LC_50_ among the organisms were not due to chance, with a highly significant *p*-value (*p* < 0.001) (Table 2).

**Figure 2:**
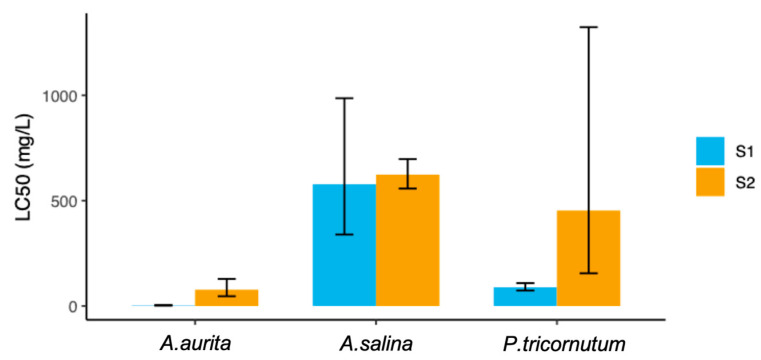
Values for all the organisms tested for each additive.

As shown in Figure 2, the LC_50_ values for three model species exposed to the additives highlight that *A. aurita* was the most sensitive to both additives, as demonstrated by the lowest LC_50_ values. In contrast, *A. salina* showed the least sensitivity, with the highest LC_50_ values among the three species. *P. tricornutum* exhibited intermediate sensitivity. These findings indicate that the jellyfish is the most vulnerable to the toxic effects of the additives, whereas the crustacean is the most resistant.

**Figure 3:**
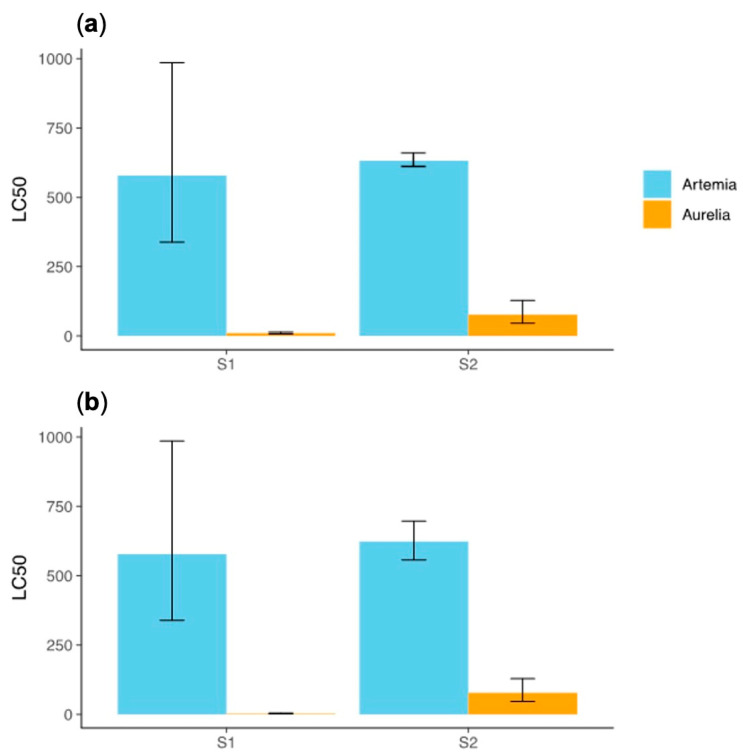
Values for *A. salina* y *A. aurita* at (**a**) 24h and (**b**) 48h for the two additives.

Comparing the effects of the compounds on the two model species, *A. salina* and *A. aurita*, at exposure times of 24 and 48 h, we found no differences between the 24 h and 48 h time points for *both* compounds in either organism. This indicates that the toxic effects of the compounds are primarily observed within the first 24 h of exposure. Therefore, the toxic effects of the compounds are established predominantly within the initial 24 h period, and prolonging exposure to 48 h does not significantly alter the toxicity (Figure 3).

However, the LC_50_ values for *P. tricornutum* were not compared to the 24 and 48 h LC_50_ values of *A. salina* and *A. aurita* because the testing protocol for the algal species uses a 72 h endpoint. Consequently, the effect of time was not considered for the alga in this study. The protocol’s focus on a 72 h exposure period for algae precluded a direct comparison with the shorter exposure times used for the other species.

According to the adapted classification, the species that experienced some level of toxicity from both compounds was *A. aurita*, while compound S1 was toxic to the studied alga. In contrast, for the crustacean *A. salina*, neither of the two compounds was toxic (Table 2 and Table 4).

**Figure 4:**
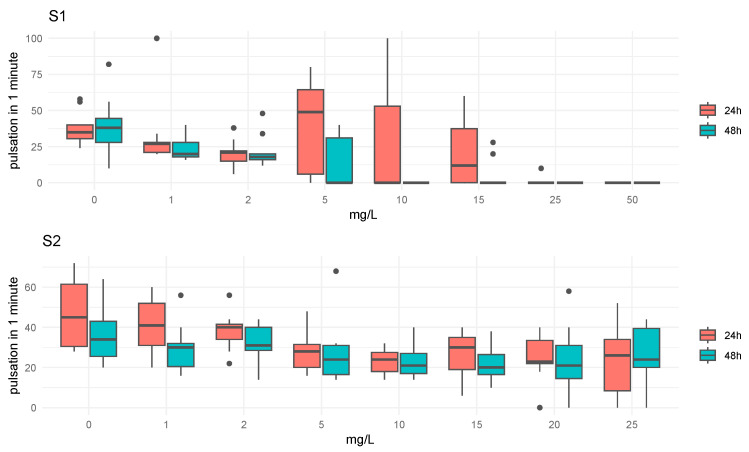
Pulsation data for each compound (S1 and S2) at both exposure times (24 h and 48 h).

The analysis of the pulsation rate (PR) in relation to each concentration and exposure time for compounds S1 and S2 is illustrated in boxplot diagrams (Figure 4). For compound S1, differences in were observed between the different concentrations at both 24 h and 48 h of exposure. The medians of the PR exhibit a decreasing trend with increasing concentration, reaching minimum values for concentrations above 5 mg/L. Similarly, for compound S2, notable differences were detected between various concentrations over the two exposure times, with the PR generally declining as concentration increased. The ANOVA test results recorded in Table 5 indicate that both concentration and time had significant main effects. Specifically, the concentration factor showed a highly significant effect (F = 13.059, *p* < 0.001), suggesting that variations in concentration lad to substantial differences in the outcome. Similarly, time had a significant impact (F = 14.133, *p* < 0.001), indicating that changes over time significantly affected the dependent variable. However, the interaction between concentration and time was not significant (F = 0.954, *p* = 0.472), implying that the combined effect of these two factors did not differ significantly from the effects of each factor individually. This analysis underscored the importance of both concentration and time in influencing the results, while suggesting that their interaction did not contribute additional variance in the dependent variable.

## 4. Discussion

The compound S1 is recognized for its established applications as a food additive, cleaning product, and fragrance ingredient. Currently, the compound is classified as toxic upon inhalation and ingestion in humans, with hazard codes H322 and H302 [34]. Hazard codes H302 and H322 correspond to specific hazard statements under the Globally Harmonized System (GHS) of Classification and Labelling of Chemicals. Hazard code H302 is associated with the phrase “May be harmful if swallowed,” indicating that the substance or mixture may pose a health risk if ingested. Hazard code H322 corresponds to the phrase “Toxic if inhaled,” highlighting the potential health hazard that may result from inhalation exposure. These codes are part of a standardized system in which each hazard statement is identified by a code consisting of the letter H followed by three digits. The codes serve as reference identifiers for hazard statements, but the full phrases should appear on labels and safety data sheets to ensure clear and effective communication of the associated risks. Despite this classification, S1 is undergoing registration under the ecotoxicity section of the REACH regulation due to a lack of sufficient and conclusive information for a definitive classification and authorization. In our study, the tests conducted suggested that S1 could be classified as “Low toxic” to *P. tricornutum* and “Medium-toxic” to *A. aurita*. However, *A. salina* was not affected by this compound. These results highlight that the toxicity of S1 is highly dependent on the susceptibility of individual species, suggesting we may be underestimating the toxicity of this additive if it only is tested with the more resistant organisms. For small mammals such as rats and guinea pigs, the LD_50_ ranges from 980 to 1300 mg/L, which is higher than the concentrations recorded for invertebrates [35]. As other authors have noted in their works, our findings support the need for more comprehensive ecotoxicity studies assessing organisms from different trophic levels within the food chain [36]. Following the previous mentioned idea, there is a possibility that we underestimated the toxic potential of the studied additives, which are currently present in the marine environment and ecosystems in general.

On the contrary, for compound S2 there are several studies related to its toxicity. It has demonstrated varying levels of toxicity across different organisms, and this is supported by our test results. Other researchers reported LC_50_ values of 100 mg/L and 345 mg/L in the fish species *Danio rerio* and *Leuciscus idus* [25]. For invertebrates, the mean LC_50_ value was 45.3 mg/L, while cyanobacteria exhibited a mean LC_50_ value of 200 mg/L. Notably, a separate study involving *P. tricornutum* recorded an LC_50_ value of 257.14 mg/L, which is significantly lower than our finding of 452 mg/L [8]. This substantial difference in the toxicity values for the additive suggests that there are species-specific and methodological factors influencing the outcomes.

**Figure 5:**
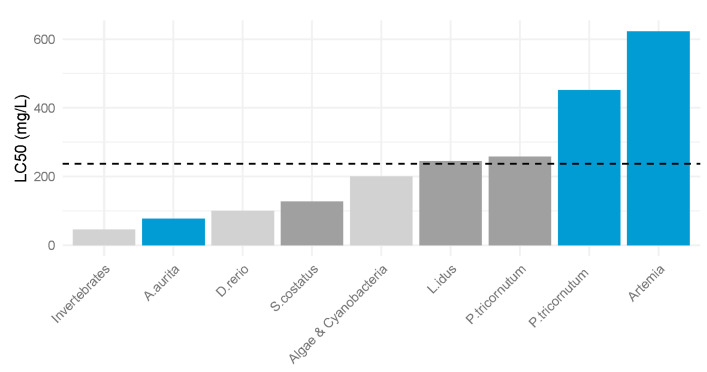
Comparison of LC_50_ values for compound S2. The grey bars represent data extracted from Dou et al. (2019) [25], while the blue bars indicate data obtained in this study. The blue bars indicate that the species has been studied in this work. The dashed lines correspond to the maximum environmental concentration values reported by Dao et al. (2014) [9].

Considering that the additive S2 is an intermediate product in the petrochemical industry, with concentrations in wastewater ranging from 114 to 237 mg/L, the importance of establishing toxicity limits becomes even more critical (Figure 5). The use of 1-phenylethanol in the perfume industry, coupled with its presence as an intermediate in the petroleum industry, increases its possibility of being present in the marine environment [9,37,38]. The discrepancies about its toxicity highlight the necessity of establishing standardized toxicity limits for better the classification and assessment of substance toxicity in marine environments. A comprehensive evaluation encompassing a diverse array of marine organisms is crucial for accurately determining the ecological risks posed by chemical compounds like S2. Following the precautionary principle, the release of this compound into the environment should be restricted or minimized until definitive results are obtained.

From a chemical standpoint, both compounds have a phenyl group attached to a carbon chain with alcohol groups as substituents. The difference between them is that S2 has the phenyl and alcohol groups attached to the same carbon atom (C1 of the ethane). On the other hand, S1 has the substituents attached to adjacent atoms (phenyl group on C1 of the propane and alcohol group on C2). The proximity of the electron-donating alcohol group to the aromatic ring may reduce the reactivity of compound S2. In contrast, in compound S1, the alcohol group, being further away from the phenyl group, has a lesser effect on the reactivity of the phenyl group. Therefore, a priori, we can reason that compound S1 is more reactive than compound S2. And thus, our data show that S1 is more toxic than S2.

The environmental risk assessment (ERA) recorded in Table 6 reveals that, for S2, the hazard quotient (HQ) values are significantly greater than one, indicating a high risk to the environment [30,31,32]. Nevertheless, we used the PEC from Dao et al. (2014) [9], who estimated the concentration from a pretreatment wastewater, and thus, we can assume a lower concentration in the marine environment. Given the potential environmental impact of these compounds, it is crucial to further study their reactivity and toxicity. Understanding these properties is essential to ensure the environmental health of our ecosystems, as even slight variations in chemical behavior could lead to significant ecological consequences. Continued research in this area will help mitigate any potential risks these compounds may pose.

In any case, both additives exhibited toxic effects on the secondary consumer model *A. aurita*. It is crucial to consider these findings for the protection of our marine ecosystems. As highlighted in several studies, there is a direct correlation between trophic diversity and the diversity of ecosystem services. Therefore, the loss of secondary consumers due to the underestimation of the effects of any pollutant or compound in our biosphere may result in the loss of intermediate trophic levels and, consequently, the degradation of ecosystem services.

To better understand the toxic effects of these additives or any other compound, we explored the potential of using the scyphozoan *A. aurita* as a model for ecotoxicology assays. As noted by Faimali et al. (2014), *A. aurita* was more sensitive to sodium dodecyl suldate than *A. salina*, *Amphibalanus*, and *Americamysis* [16]. Additionally, Mercado et al. (2023) further supported the work of Faimali by testing the potential of *A. aurita* as a new model for ecotoxicology due to its ecological relevance, broad distribution, and sensitivity to various environmental contaminants, reinforcing its value for assessing pollutant impacts on marine ecosystems [19]. In addition, our findings further support the use of *A. aurita* as an ecotoxicological model, given its greater sensitivity to the compounds tested in this study. We therefore recommend its inclusion alongside other established models to obtain more comprehensive information that can help assess the environmental impact of these substances in marine ecosystems.

## 5. Conclusions

Our study demonstrated that S1 is toxic or has medium toxicity to the alga *P. tricornutum* and the scyphozoan *A. aurita* but not to *A. salina*. In contrast, S2 is only toxic to the cnidarian and non-toxic to the other two organisms. In both cases, the scyphozoan proved to be the most sensitive organism, corroborating findings from previous studies. This suggests that *A. aurita* could serve as an important model for toxicity testing with secondary consumers, enhancing assay sensitivity and preventing the underestimation of the toxic potential of substances. Therefore, our work supports the validity of ephyras as a model for toxicity assays.

Additionally, our results highlight the urgent need to establish universal toxicity criteria to reduce the environmental impact of industrial substances that enter wastewater and subsequently the marine environment. Implementing these criteria is crucial for better protecting marine ecosystems from potentially harmful chemicals.

## Figures and Tables

**Table 1:** Chemical characteristics of each compound including water solubility.

ID	Substance	CAS	Formula	Water Solubility (mg/L)	Chemical Structure
S1	2-Methyl-1-phenylpropan-2-ol	100-86-7	C_10_H_14_O	13,538.094 (at 20 °C)	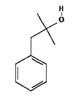
S2	1-Phenylethan-1-ol	98-85-1	C_8_H_10_O	3717.5 (at 25 °C)	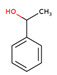

**Table 2:** LC_50_ and LC_10_ and the 95% confidence limit (C.L.) of each organism model for each tested compound.

ID	Organism	LC_50_ (mg/L)	95% C.L.	
			Lower	Upper
S1	*A. salina*	577.9	338.8	985.8
	*P. tricornutum*	89.0	72.95	108.4
	*A. aurita*	2.5	1.4	4.3
S2	*A. salina*	623.3	557.3	697.0
	*P. tricornutum*	452.9	155.1	1323.3
	*A. aurita*	77	46.3	128.2
**ID**	**Organism**	**LC_10_ (mg/L)**	**C.L. 95%**	
			**Lower**	**Upper**
S1	*A. salina*	130.7	66.6	194.0
	*P. tricornutum*	69.2	56.76	84.3
	*A. aurita*	0.29	0.17	0.51
S2	*A. salina*	140.9	77.9	254.7
	*P. tricornutum*	28.8	9.9	84.3
	*A. aurita*	22	13.2	36.7

**Table 3:** Analysis of variance for the LC_50_ of each compound.

		Df	Sum Sq	Mean Sq	F Value	Pr (>F)
S1	Organism	2	1926.530	963.265	3295.947	$<2\times 10^{-16}$
	Residuals	27	8	0		
S2	Organism	2	1562.053	781.027	5199.973	$<2\times 10^{-16}$
	Residuals	27	4	0		

**Table 4:** Toxicity classification of each additive according each LC50 and the categorical criteria used. Color codes for the different toxic categories: cat 1, highly toxic substances with E(L)C50 ≤ 1 mg/L (red); cat 2 (orange), from >1 to ≤10 mg/L (medium toxicity); cat 3 (yellow), from >10 to ≤100 mg/L (low toxicity); and cat 4 for non-toxic substances (green) with E(L)C50 > 100 mg/L.

ID	Compound	CL50—48-h *A. salina*	CL50—72-h *P. tricornutum*	CL50—48-h *A. aurita*
S1	2-Methyl-1- phenylpropan-2-ol	Non-toxic	Low toxicity	Medium toxicity
S2	1-Phenylethan-1-ol	Non-toxic	Non-toxic	Low toxicity

**Table 5:** Analysis of variance for the pulsation rate.

	Df	Sum Sq	Mean Sq	F Value	Pr(>F)
Concentration	8	29,191	3649	13.059	$<4.08\times 10^{-16}$
Exposure time	1	3949	3949	14.133	0.000205
Concentration: time	8	2134	267		

**Table 6:** Data from ERA analysis.

Organism	LC_10_ (mg/L)	Average PEC (mg/L) *	PNEC (mg/L) **	HQ (PEC/PNEC)
*A. salina*	140.9	175.5	0.1409	1266.85
*P. tricornutum*	28.8	175.5	0.0288	6232.63
*A. aurita*	22	175.5	0.022	8204.54

* Data obtained from Dao et al. (2014) [9]: 237–114 mg/L for S2. ** PNEC calculated using assessment factor of 1000.

## Data Availability

O.A.-S. (Ph.D.), on behalf of the rest of the coauthors, with this document, warrantees and signs that the datasets generated and used during the current study are available from the corresponding author upon reasonable request.

## Supplementary Materials

The following supporting information can be downloaded at: https://www.mdpi.com/article/10.3390/toxics13070572/s1, Figure S1: Dose-response curve for 2-Methyl-1-phenylpropan-2-ol (S1) and Phenylethan-1-ol (S2). (A) for the *Aurelia aurita* jellyfish, (B) for *Artemia salina* microcrustacean and (C) for the *Phaeodactylum tricornutum* algae. The confidence level associated to the regression is showed in form of shadow. Table S1: Percentages of decrease of the chemicals’ concentration along the assay respect to the nominal concentration. In parenthesis the higher concentration tested in any assay. Video S1: Video illustrating pulsation of *Aurelia aurita* ephyrae after the strobilation.
