# Supplementary material for: Aurelia aurita as a Model for Ecotoxicologically Assessing Food Additives: 2-Methyl-1-phenylpropan-2-ol and 1-Phenylethan-1-ol
Source: Toxics. 2025 Jul 7;13(7):572. doi: 10.3390/toxics13070572 (PMC12300291; doi:10.3390/toxics13070572)
Supplement: Supplementary file 1 [file toxics-13-00572-s001.zip › Supplementary material.pdf]

Supplementary material: *Aurelia aurita* as a model for assessing food additives: Comparative Results Across Trophic Levels

Table S1: Percentages of decrease of the chemicals' concentration along the assay respect to the nominal concentration. In parenthesis the higher concentration tested in any assay.

Exposure time (h)	Bioassay		
	Aurelia aurita	*Artemia salina*	*Phaeodactylum tricornutum*
0	100% (50 mg/L)	100% (1000 mg/L)	100% (500 mg/L)
24	98.2%	97.4%	96.8%
48	96.3%	96.1%	95.5%

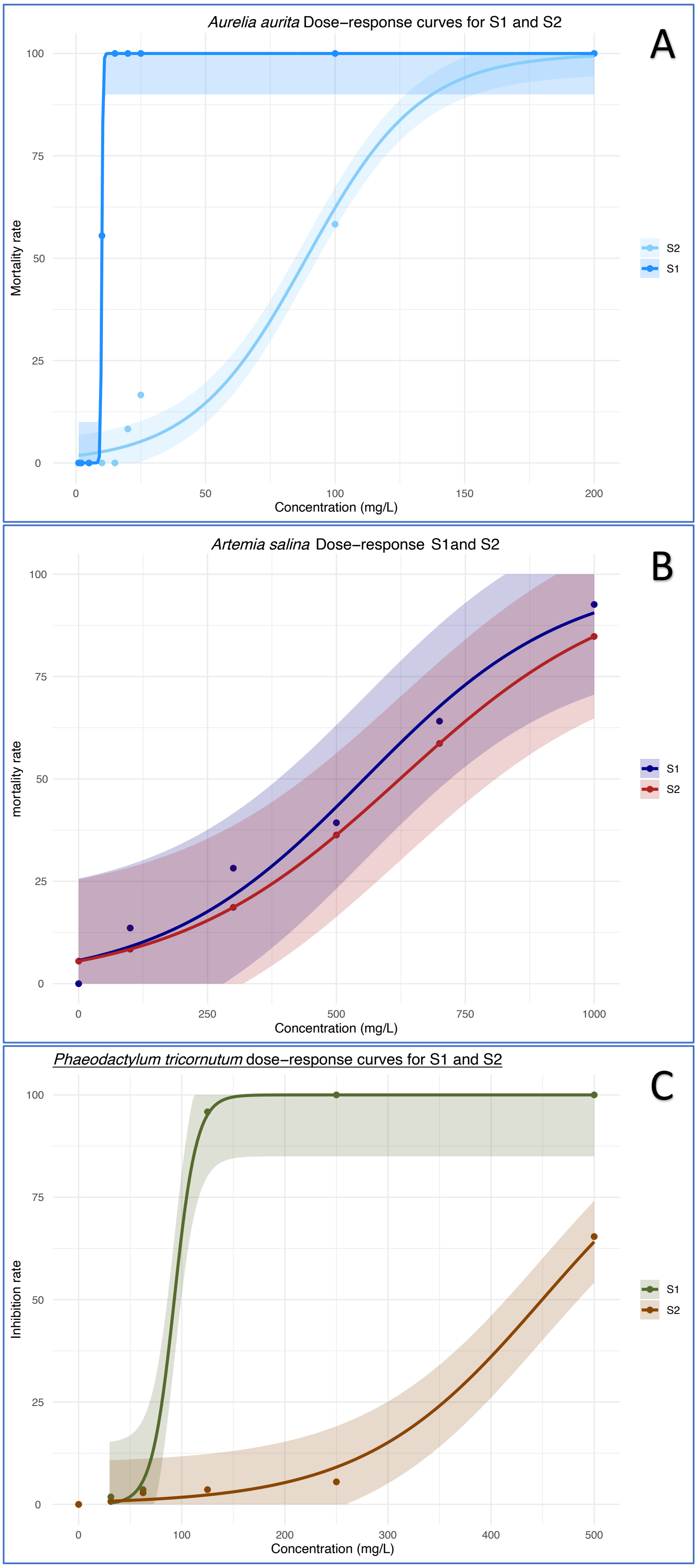

Figure S1. Dose-response curve for 2-Methyl-1-phenylpropan-2-ol (S1) and Phenylethan-1-ol (S2). (A) for the *Aurelia aurita* jellyfish, (B) for *Artemia salina* microcrustacean and (C) for the *Phaeodactylum tricornutum* algae. The confidence level associated to the regression is showed in form of shadow.

Video S1. Video illustrating pulsation of *Aurelia aurita* ephyrae after the strobilation

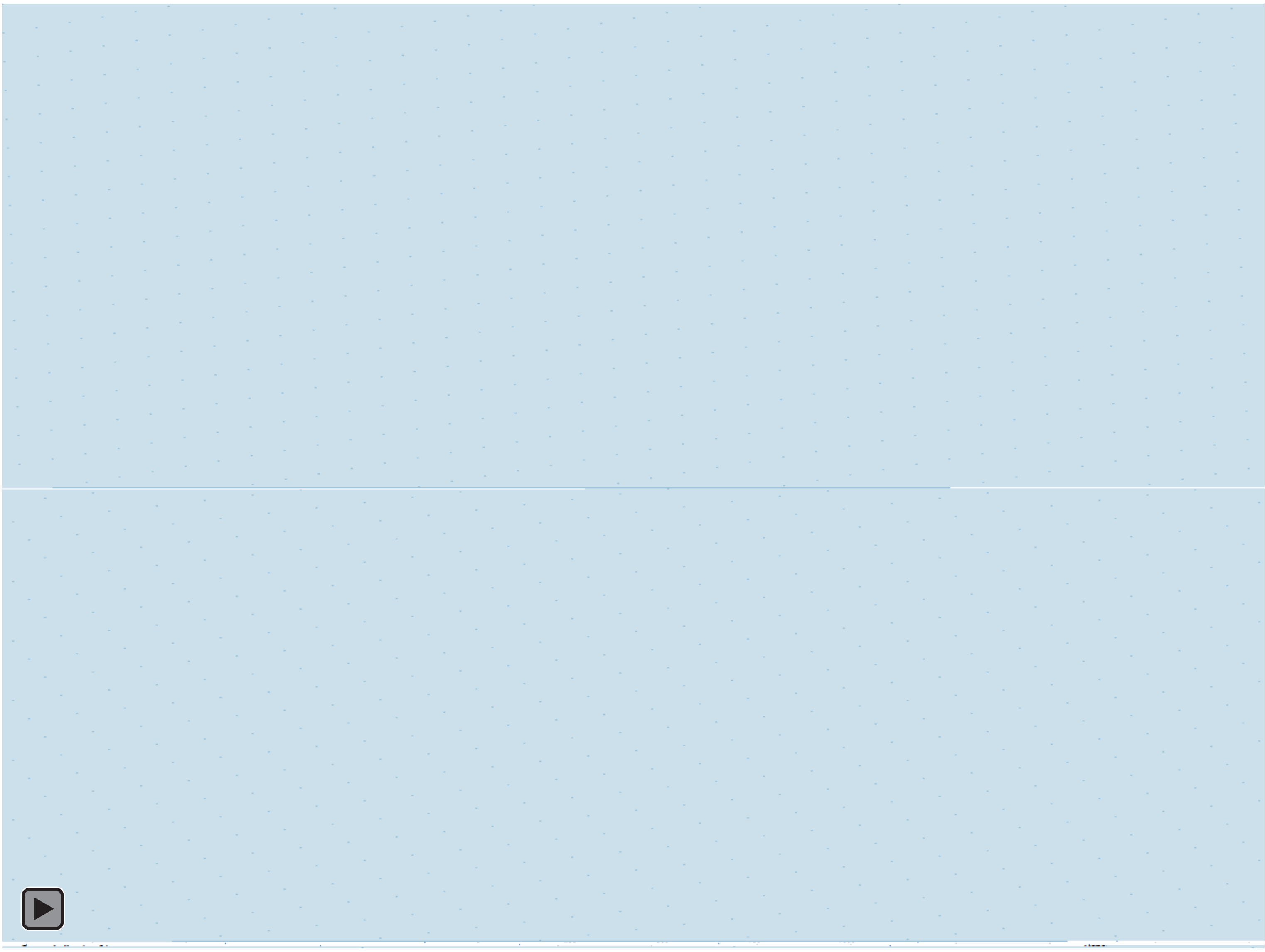

Link to video
